# Genome-wide identification, isolation and expression analysis of auxin response factor (*ARF*) gene family in sweet orange (*Citrus sinensis*)

**DOI:** 10.3389/fpls.2015.00119

**Published:** 2015-03-30

**Authors:** Si-Bei Li, Wei-Zhi OuYang, Xiao-Jin Hou, Liang-Liang Xie, Chun-Gen Hu, Jin-Zhi Zhang

**Affiliations:** ^1^Key Laboratory of Horticultural Plant Biology, Ministry of Education, College of Horticulture and Forestry Science, Huazhong Agricultural UniversityWuhan, China; ^2^College of Plant Sciences and Technology, Huazhong Agricultural UniversityWuhan, China

**Keywords:** ARF gene, auxin, citrus, expression analysis, phylogenetic analysis, sweet orange

## Abstract

Auxin response factors (ARFs) are an important family of proteins in auxin-mediated response, with key roles in various physiological and biochemical processes. To date, a genome-wide overview of the *ARF* gene family in citrus was not available. A systematic analysis of this gene family in citrus was begun by carrying out a genome-wide search for the homologs of ARFs. A total of 19 nonredundant *ARF* genes (*CiARF*) were found and validated from the sweet orange. A comprehensive overview of the *CiARF*s was undertaken, including the gene structures, phylogenetic analysis, chromosome locations, conserved motifs of proteins, and *cis*-elements in promoters of CiARF. Furthermore, expression profiling using real-time PCR revealed many *CiARF* genes, albeit with different patterns depending on types of tissues and/or developmental stages. Comprehensive expression analysis of these genes was also performed under two hormone treatments using real-time PCR. Indole-3-acetic acid (IAA) and N-1-napthylphthalamic acid (NPA) treatment experiments revealed differential up-regulation and down-regulation, respectively, of the 19 citrus *ARF* genes in the callus of sweet orange. Our comprehensive analysis of ARF genes further elucidates the roles of *CiARF* family members during citrus growth and development process.

## Introduction

Auxin plays a central role in controlling plant developmental and physiological processes, including embryogenesis, apical dominance, vascular elongation, flowering, fruit development, and lateral root initiation (Woodward and Bartel, 2005; Fleming, 2006). Previous studies indicated that two types of transcription factor families are necessary to modulate / regulate the expression of auxin response genes during growth and development process: auxin response factors (ARFs) and Aux/IAA (Guilfoyle and Hagen, 2007). Most ARF proteins consist of an N-terminal B3-type DNA binding domain, a variable middle region that may function as an activation or repression domain, and two C-terminal Aux/IAA domains, which are involved in protein–protein interaction by dimerizing with Aux/IAA family genes (Ulmasov et al., 1999b; Hagen and Guilfoyle, 2002). ARF proteins can either activate or repress auxin responsive gene transcription depend on the amino acid composition of their variable internal region (Ulmasov et al., 1999a). Irrespective of auxin status, ARF proteins are competent to bind to auxin responsive *cis*-elements present upstream of the coding sequence of auxin responsive genes. Activation domains of ARFs are rich in leucine, serine, and glutamine residues, while the repression domains are rich in serine, proline, glycine, threonine, and serine residues (Ulmasov et al., 1997, 1999a).

Recent advances have elucidated the regulation of *ARF* gene expression. Classical genetic approaches have enabled identifying *ARF* gene functions in the growth and development of model plants based on the characterization of gain-of-function mutants such as *Arabidopsis*. For example, mutations to *ARF* genes resulted in changes in the embryo axis formation and vascular strands (*AtARF5*), suppression of hypocotyls bending and hookless phenotype (*AtARF1*/*2*), increased weight and size of seeds (*AtARF2*), abnormal floral organs and leaves (*AtARF3*/*4*), impaired hypocotyl response to blue light and auxin sensitivity (*AtARF7*), and changed auxin homeostasis (*AtARF8*) (Ellis et al., 2005; Nishimura et al., 2005; Fukaki et al., 2006; Schruff et al., 2006; Guilfoyle and Hagen, 2007; Finet et al., 2010). Since cloning of *AtARF1*, the first *ARF* gene from *Arabidopsis*, 22 other members of this family from *Arabidopsis* (Ulmasov et al., 1997), 25 from rice (Wang et al., 2007), 31 from maize (Xing et al., 2011), and 39 from poplar (Kalluri et al., 2007) have been identified. Despite the importance of ARF genes in multiple aspects of plant growth and development, these gene families remain largely uncharacterized in perennial species, and the degree of conservation of gene families between herbaceous and perennial plants is unknown. Furthermore, the regulation mechanisms of *ARF* are not completely understood in perennial plants and much remains to be learned about their roles in the contexts of other plants. Therefore, identification of *ARF* gene families from perennial plants is a necessary step in formulating better hypotheses related to growth and development.

Citrus is an important species of woody perennial trees grown around the world for the production of fresh fruit and juice among other products (Tan and Swain, 2007). Auxin plays a pivotal role in various aspects of citrus growth and in developmental processes such as flowering, fruit set, fruit ripening, tissue differentiation, and morphogenesis (Mendes et al., 2011; Mesejo et al., 2012). To date, no systematic investigations of *ARF* family genes have been reported in citrus. The recent release of the sweet orange genome has provided a reference for testing inferences about auxin signal transduction events previously obtained through studies of *Arabidopsis* (Xu et al., 2013). In the current work, we summarize findings from bioinformatics studies to identify the total 19 *CiARF* genes, predict the protein domains, and assess the extent of conservation and divergence between citrus and *Arabidopsis*. We have also used real-time PCR studies to systematically characterize the expression of the entire gene family in different tissue organs of sweet orange to obtain information about each of the family members during citrus growth and development process. Meanwhile, the expression profiles of the *CiARF* genes were also analyzed under indole-3-acetic acid (IAA) and N-1-napthylphthalamic acid (NPA) treatment conditions. Such a comprehensive analysis may prove fundamental to understanding the diverse roles of *ARF* genes in citrus growth and development. These results provide a solid base for future functional genomic studies of the *ARF* gene family in citrus.

## Materials and methods

### Identification and classification of *ARF* genes

The sweet orange genome was downloaded from the *Citrus sinensis* Annotation Project (CAP; http://citrus.hzau.edu.cn/orange/). The BLASTP search was used to identify the members of the *ARF* gene family of sweet orange. Two BLASTP methods were adopted to search for the *ARF* genes of sweet orange and to identify the maximum number of genes. Firstly, all publicly known *Arabidopsis ARF* genes (*AtARF1* to *AtARF23*) were used in the initial protein queries on website of CAP (http://citrus.hzau.edu.cn/cgi-bin/orange/blast) and candidate genes were identified based on a BLASTP search at the score value of ≥100 and *e*-value ≤e^−10^ (Kumar et al., 2011). Secondly, key words “auxin response factor” and “B3 DNA binding domain (IPR003340)” were used as queries to search against sweet orange Genome (http://citrus.hzau.edu.cn/cgi-bin/orange/search). Next, the Pfam database was used to determine if each candidate *ARF* sequence was a member of the *ARF* gene family. To exclude overlapping genes, all candidate *ARF* genes were aligned using ClustalW (Thompson et al., 1994; Hou et al., 2014) and checked manually. All non-overlapping *ARF* genes were used for further analysis. Information about coding sequence, full-length sequence, and amino acid sequence was also obtained for each gene from CAP by BLAST program (Xu et al., 2013).

### Analysis of conserved motif and subcellular localization

To examine the structural divergence among the *CiARF* genes, the conserved motif was investigated in the encoded ARF proteins. Their complete amino acid sequences were subjected to MEME analysis online (http://meme.nbcr.net/meme/; Bailey and Elkan, 1995; Hou et al., 2014) with the following parameters: (1) optimum motif width was set from 6 to 200; and (2) the maximum number of motifs was set to identify 15 motifs. The subcellular localization of CiARF proteins were predicted by subCELlular LOcalization predictor (CELLO v.2.5; http://cello.life.nctu.edu.tw/, Yu et al., 2006).

### Gene structure and chromosomal locations of *CiARF* genes

To illustrate the structure of intron and exon of *CiARF* genes, gene structure display server (GSDS) program (Guo et al., 2007) was used to draw the gene structure according to the full-length genome and coding sequence from the CAP database (Xu et al., 2013). To determine their physical location, the starting positions of all *CiARF* genes on each chromosome were confirmed based on a local database of the complete sequence of the sweet orange genome by BlastN searching. MapInspect software was used to draw the location images of *CiARF* genes (http://www.plantbreeding.wur.nl/uk/software_mapinspect.html; Zhao et al., 2011).

### Phylogenetic and promoter motifs analysis of *CiARF* genes

All conserved domains were investigated by multiple alignment analyses using ClustalW (Thompson et al., 1994). Phylogenetic analysis for all complete CiARF protein sequences was performed using MEGA 6 program by the neighbor-joining method, the parameters was set according to the method of (Tamura et al., 2013). Bootstrap analysis was performed by using 1000 replicates. Protein sequences and open reading frames (ORFs) of the gene pairs were also aligned by MEGA 6 program, respectively.

The *cis*-motifs of CiARF promoters were identified in a 2-kb upstream sequence from translational initiation codon of *CiARF* genes using the MEME program. This program was used to search the five best *cis*-motif consensus patterns of 8–50 bases width, with *E*-value <0.01. Graphs of motifs were plotted according to their position within the region using WebLogo tool (http://weblogo.berkeley.edu/logo.cgi). Discovered motifs were analyzed using PLACE (Lescot et al., 2002).

### Plant growth and treatments

The tissues and organs from sweet orange were collected in the experiment fields of the National Citrus Breeding Center at Huazhong Agricultural University. To analyze tissue or organ-specific expression of *CiARF* genes, mature leaves, stems from spring shoots (about 45 days), lateral roots, flowers at full bloom and whole fruits at 30 days after flowering (DAF) were collected from adult plants. Meanwhile, the various floral organs (torus, petal, stamen, and ovary) were collected from the flower buds (about 3 days before opening). To analyze the expression pattern during the fruit ripening process, sweet orange fruit samples were also collected at 170 DAF (fully deep green: stage 1), 200 DAF (yellowing started: stage 2), 230 DAF (partly green in nearly fully yellow: stage 3), and 250 DAF (fully golden yellow: stage 4).

Callus of sweet orange was maintained *in vitro* for 2-week intervals on callus growth medium containing Murashige and Skoog medium, 3% sucrose, 10 μM naphthalene acetic acid (NAA), 1 μM N 6-benzylaminopurine, and 0.7% agar (pH 5.8) in the dark at 25°C. After subculture for four cycles, 2-week-old calluses were used for two different experiments. In experiment I, calluses were cultured on callus propagation medium supplemented with 1, 5, 10, and 100 μM IAA, respectively. In experiment II, calluses were cultured on callus propagation medium supplemented with 1, 5, 10, and 100 μM NPA, respectively. Callus samples were collected at 0, 6, and 12 h. Each experiment was repeated three times. All the samples were stored at −80°C. Total RNA was isolated according to a previous protocol (Zhang et al., 2008).

### Expression analysis of *CiARF* genes by real-time PCR

Total RNA (3 μg) was treated with 3 U of DNase (Promega) to remove DNA contamination and used in first-strand synthesis with an oligo (dT) primer (20-mer) and reverse transcriptase according to the manufacturer's instructions. The *β-actin* gene of citrus was designed to act as an internal control. The primers were designed with the Primer Express software and tested to ensure amplification of single discrete bands with no primer-dimers. Product size was 150–200 bp. Primer sequences are shown in detail in the Supplementary Table S1. The expression level of *CiARF* genes was measured by real-time PCR using the SYBR Green PCR Master Mix (Roche Applied Science) as described previously (Hou et al., 2014). The data were processed using One-Way analysis of variance (ANOVA), and statistical differences were compared based on Student's *t*-test, taking *P* < 0.05 as ^*^and *P* < 0.01 as ^**^. Three biologic repeats and four mechanical repetitions were assayed in this study, giving similar trends. Data from one biologic repeat are presented.

## Results

### Identification of *CiARF* genes

To identify the *ARF* genes in sweet orange, two BLAST approaches were employed for the mining of all putative *ARF* members in the sweet orange genome. By the two approaches, more than 100 *ARF* genes were identified from the sweet orange genome. Because the sweet orange genome was sequenced with a whole-genome shotgun strategy, some of these *ARF* genes may be redundant even though they were located on distinct scaffolds. By removal of the sequence redundancies and alternative splice forms of the same gene, a total of 19 potential ARF proteins were identified as being associated with *CiARF* genes (Table 1). The nomenclature system for *CiARF* in the present study provisionally uses the names *CiARF1* to *CiARF9* and *CiARF16* to *CiARF19* to distinguish each of the *ARF* genes based on the homology between *AtARF* and *CiARF* genes. Because the other five homologous genes were not found in *Arabidopsis*, naming them based on homolog proteins in *Arabidopsis* was not possible. Sequence analysis of these five genes indicated that the similarity with the remaining *Arabidopsis ARF* genes was quite low, so these genes were named from *CiARF10* to *CiARF15* according to their position from the top to the bottom on citrus chromosomes 1–9 (Table 1). The ORF length of *CiARF* genes varied from 390 bp (*CiARF11*) to 3375 bp (*CiARF19*), encoding polypeptides of 129–1124 amino acids, with a predicted molecular mass range of 14.67–125.76 kDa; the theoretical pI ranged from 5.11 to 10.24 (Table 1). Pair-wise analysis of CiARF proteins indicated that the overall identity fell in a range from 53.24% (between CiARF13 and CiARF18) to 89.23% (between CiARF11 and CiARF19).

**Table 1:** ***CiARF* genes encoding ARF proteins along with their molecular details**.

**Gene**	**Gene ID**	**No. exon**	**Protein length**	**Mw (kDa)**	**pI**	**Location**	**Domain**	**Homologous**	***E*-value**	**Similarity (%)**	**Localization**
*CiARF1*	Cs03g01570.1	14	688	76.5	5.82	Chr3	DBD, ARF, CTD	AtARF1	1e^−114^	80	Nuclear
*CiARF2*	Cs7g19770.1	14	846	94.5	5.94	Chr7	DBD, ARF, CTD	AtARF2	1e^−108^	80	Nuclear
*CiARF3*	Cs3g05470.1	10	481	53.4	7.62	Chr3	DBD, ARF	AtARF3	2e^−26^	80	Nuclear
*CiARF4*	Cs8g16930.1	12	808	89.7	6.73	Chr8	DBD, ARF, CTD	AtARF4	1e^−74^	79	Nuclear
*CiARF5*	Cs3g25860.1	14	946	105	5.48	Chr3	DBD, ARF, CTD	AtARF5	6e^−74^	78	Nuclear
*CiARF6*	Cs2g09440.2	14	899	99.4	6.19	Chr2	DBD, ARF, CTD	AtARF6	1e^−154^	81	Nuclear
*CiARF7*	Cs4g04520.1	13	1053	116	6.44	Chr4	DBD, ARF, CTD	AtARF7	6e^−22^	84	Nuclear
*CiARF8*	Cs6g16030.2	14	797	89.1	5.87	Chr6	DBD, ARF, CTD	AtARF8	e^−161^	81	Nuclear
*CiARF9*	Cs5g01980.1	14	690	77.4	6.4	Chr5	DBD, ARF, CTD	AtARF9	2e^−57^	79	Nuclear
*CiARF10*	Cs2g15130.1	14	898	99.1	6.04	Chr2	DBD, ARF, CTD	AtARF6	e^−134^	80	Nuclear
*CiARF12*	Cs5g32400.1	14	714	79.2	6.39	Chr5	DBD, ARF, CTD	AtARF2	1e^−6^	71	Nuclear
*CiARF13*	Cs6g11800.1	3	703	77.6	6.54	Chr6	DBD, ARF, CTD	AtARF16	6e^−18^	79	Nuclear
*CiARF14*	Cs7g02210.1	12	783	87.9	5.11	Chr7	DBD, ARF	AtARF4	4e^−7^	69	Nuclear
*CiARF15*	Cs8g16440.1	4	724	79.6	6.99	Chr8	DBD, ARF, CTD	AtARF16	2e^−21^	81	Nuclear
*CiARF16*	Cs7g25670.2	3	694	76	6.27	Chr7	DBD, ARF, CTD	AtARF16	1e^−59^	84	Nuclear
*CiARF17*	Cs3g18940.1	2	574	63.5	5.89	Chr3	DBD, ARF	AtARF17	7e^−8^	69	Chloroplast
*CiARF18*	orange1.1t00508.1	14	699	77.3	6.03	ChrUn	DBD, ARF, CTD	AtARF18	9e^−20^	79	Nuclear
*CiARF19*	Cs4g07020.1	13	1124	126	6.23	Chr4	DBD, ARF, CTD	AtARF19	1e^−167^	81	Nuclear

A previous study showed that *AtARF23* is a partial-length gene with a stop codon in its B3 DNA-binding domain (Guilfoyle and Hagen, 2007); a truncated gene (*CiARF11*) was also identified in the sweet orange genome. Thus, CiARF gene may represent putative pseudogenes or incorrect annotations, and manual reannotation was performed to correct using online web server FGENESH (http://linux1.softberry.com/berry.phtml). However, *CiARF11* still encoding only truncated protein was excluded from further analysis. In addition, the subcellular localization of CiARF proteins was also predicted using subCELlular LOcalization predictor (Table 1). The predicted locations of 17 CiARF proteins were found to be nuclear localized. The remaining one member of CiARF proteins was predicted to be localized in chloroplast (CiARF17).

### Comparative phylogenetic, gene structure and GO annotation of *CiARF* genes

**Figure 1:**
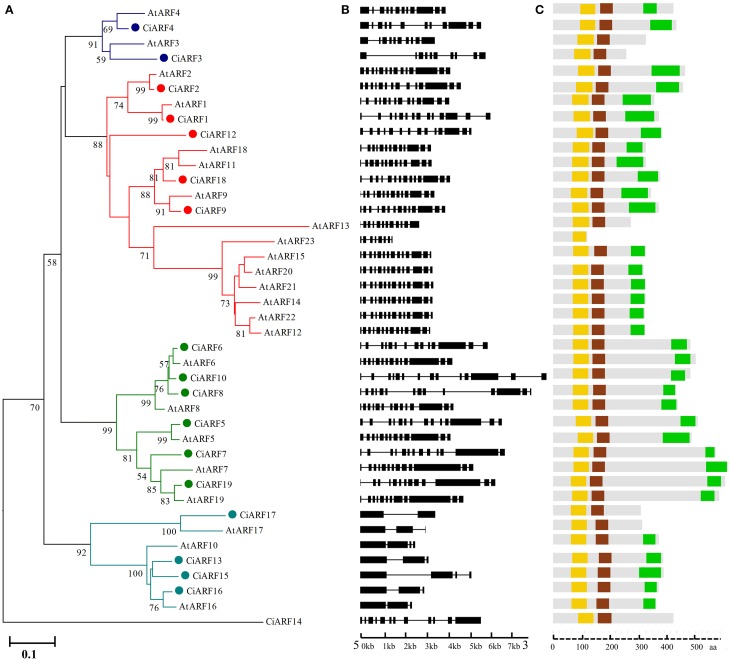
**Phylogenetic relationship, gene structure, and conserved motif analysis of the sweet orange *ARF* gene family**. **(A)** Phylogenetic analysis of ARF proteins between sweet orange and *Arabidopsis*. A total of 18 CiARF proteins from sweet orange and 23 from *Arabidopsis* were used to construct the neighbor-joining tree. **(B)** Gene structure analysis of *CiARF* and *AtARF* genes according to the phylogenetic relationship. Lengths of exons and introns of each *CiARF* gene are displayed proportionally. The boxes represent exons; black lines represent introns. **(C)** Conserved motif analysis of ARF proteins from sweet orange and *Arabidopsis* according to the phylogenetic relationship. The DBD, ARF, and CTD domains are marked in orange, brown, and green, respectively.

To explore the phylogenetic relationship of ARF proteins between *Arabidopsis* and sweet orange, a phylogenetic tree comprising 41 ARF family members from *Arabidopsis* (23 genes) and sweet orange (18 genes) was constructed. The phylogenetic distribution revealed that *ARF* genes group into five major classes, class I, II, III, IV, and V, with well-supported bootstrap values (Figure 1A). Four members were clustered in class I (with two members from sweet orange), 18 members were clustered in class II (five from sweet orange), seven members were clustered in class III (six from sweet orange), and seven members were clustered to class IV (four from sweet orange). Notably, class V had no representative in *Arabidopsis* and only contained one member from sweet orange (*CiARF14*) (Figure 1A). To understand the structural components of *CiARF* genes, the exon and intron organization of the genes was obtained by comparing the cDNA sequences with the corresponding genomic DNA sequences (Figure 1B). The coding sequences of the entire *CiARF* family were disrupted by introns, and the number of exons varied from 1 to 14 (Figure 1B). In general, the most closely related members from the same subfamilies shared similar exon/intron structure with regard to intron number and exon length.

To investigate the biological processes possibly regulated by the 18 *CiARF* genes, GO annotation of these genes was performed by Blast2GO. Figure S1 summarizes the categorization of these *CiARF* genes according to the biological process, cellular component, and molecular function in which they are implicated. Based on biological process (Figure S1A), 18 *CiARF* genes were classified into five categories: regulation of transcription (12 genes), auxin mediated signaling pathway (12 genes), flower development (6 genes), phyllome development (6 genes), and post-embryonic organ development (5 genes). These results suggest that *CiARF* genes are involved in a broad range of citrus physiological functions, and it will be an interesting challenge to link the specific functions with individual *CiARF* genes in citrus. Categories based on molecular function placed the *CiARF* genes into three groups (Figure S1B): DNA binding (16 genes), protein dimerization activity (13 genes), and sequence-specific DNA binding transcription factor activity (six genes). Based on cellular components, these CiARF proteins were localized to the nucleus in sweet orange (Figure S1C), consistent with the prediction using the localization predictor software.

### Domains and motifs characterization of *CiARF* proteins

Multiple alignment results indicated that most CiARF proteins contained three characteristic regions (Figure 1C). All CiARF proteins had a highly conserved region of about 100 amino acid residues in their N-terminal portion corresponding to the DNA-binding domain. The middle region of ARFs have been reported to function as activation or repression domains and C-terminal Aux/IAA domains (Ulmasov et al., 1999a). To further investigate the characteristic regions of CiARF proteins, online MEME was employed to analyze motif distributions in 18 CiARF proteins, with 15 individual motifs isolated. As predicted, most of the close homologs from the phylogenetic tree had common motif compositions (Figure S2), suggesting functional similarities within the same subfamily. However, the biological significance of most of the putative motifs remains uncharacterized because they lack homologs within the Pfam and SMART databases (Figure S2). Three domains of CiARF proteins were divided into 11 motifs. Motif 1 constituted the DBD. The ARF domain consisted of motifs 3, 5, 8, 9, and 10. The CTD corresponded to motifs 6, 7, and 10, respectively. Motifs 1, 2, and 4 were found in all 19 CiARF proteins (Figure S2).

**Figure 2:**
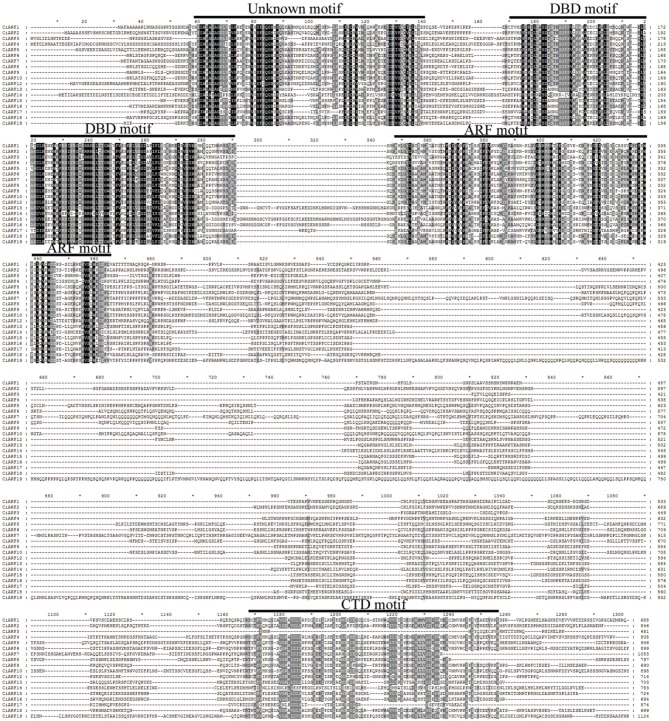
**Multiple sequence alignment of the CiARF proteins obtained with ClustalX** Conserved domains of CiARF proteins are marked by the overline.

Transfection assays with plant protoplasts indicated that AtARF1, 2, 3, 4, and 9 act as repressors (Ulmasov et al., 1999a; Tiwari et al., 2003); AtARF1 contains a middle region rich in proline, serine, and threonine. AtARF5, 6, 7, 8, and 19, with middle regions rich in glutamine, are activators (Tiwari et al., 2001; Wang et al., 2005). The detailed sequence analysis of all 19 deduced CiARF proteins identified proline, serine, and threonine-rich middle regions in CiARF1, 2, 3, 4, and 9, indicating that these genes are likely to act as repressors (Figure 2). Glutamine-rich regions were found in CiARF5, 6, 7, 10, 18, and 19, implying that these genes are probable transcriptional activators in citrus development process (Figure 2).

### Chromosomal distribution of the *CiARF* genes

**Figure 3:**
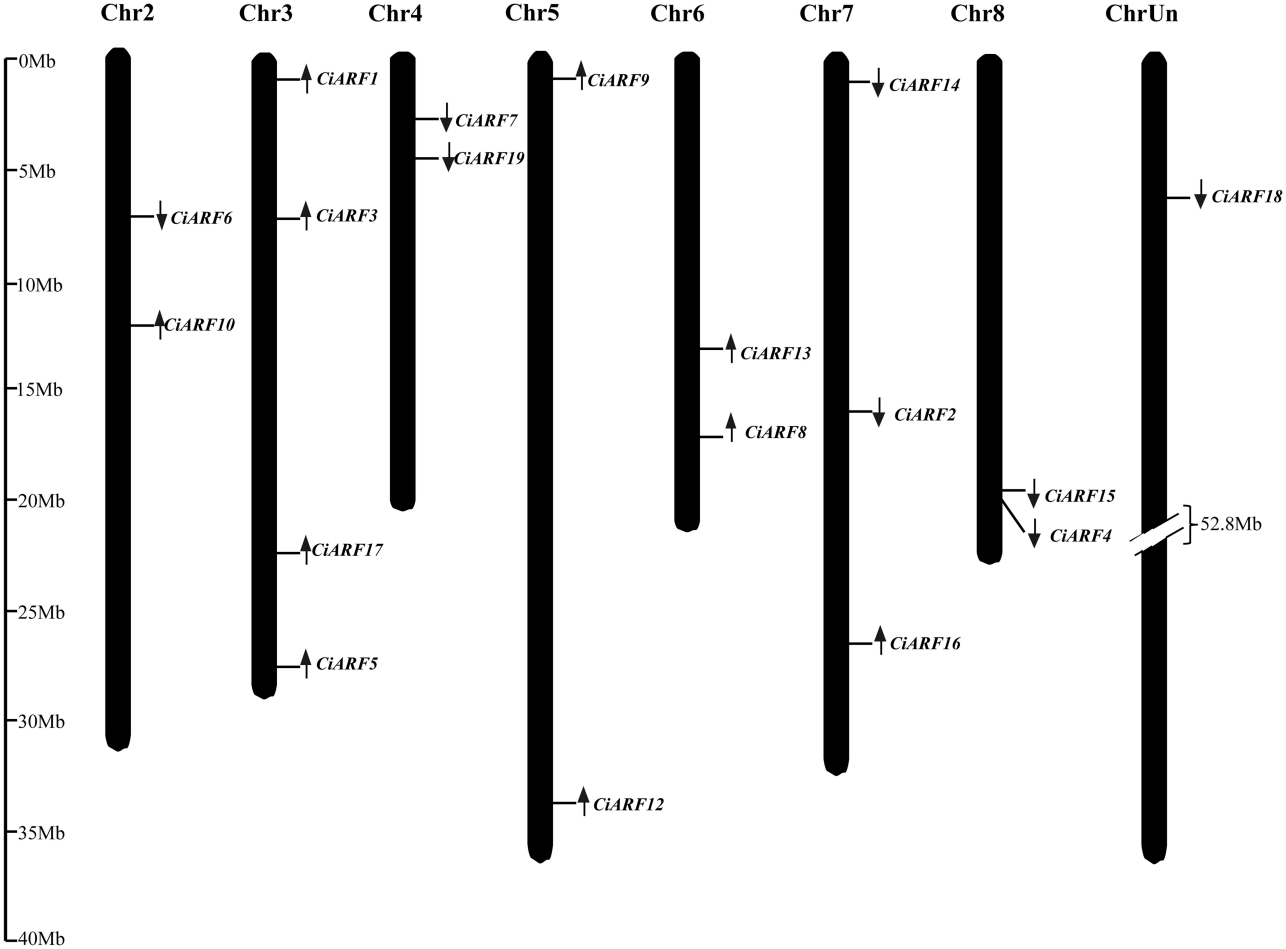
**Distribution of *CiARF* genes in sweet orange genome; the chromosomal position of each *CiARF* gene is mapped according to the sweet orange genome**. The chromosome number is indicated at the top of each chromosome. The scale is in megabases (Mb).

The chromosomal locations and transcription directions of 18 CiARF genes were demonstrated on sweet orange chromosome pseudomolecules available at CAP (http://citrus.hzau.edu.cn/orange/) using BLASTN analysis. These genes were distributed over seven of the nine sweet orange chromosomes; none of the genes occurred on chromosomes 1 and 9. Each of the chromosomes with CiARF genes had one to four of the genes. The largest number of CiARF genes was located on chromosome 3 (four), followed by chromosome 7 (three), and two genes each were located on chromosomes 2, 4, 5, 6, and 8 (Figure 3). In addition, the chromosomal location for one CiARF gene was not defined because the physical map for sweet orange was incomplete (Figure 3).

### Promoter *cis*-element and *cis*-motif prediction of *CiARF* genes

To analyze *cis*-elements in the promoter regions of *CiARF* genes, the 2.0 kb of genomic DNA sequences upstream of the start codon was first analyzed by the ORF Finder tool. These putative promoters were then used to query the GenBank database by BLAST. Results indicated that the surveyed 2.0-kb sequences were not coding sequences. A search of the PLACE database with the 2.0-kb upstream regions of the 19 *ARF* genes as queries yielded a large number of putative *cis*-elements more than 4 bp in length. Among these putative *cis*-elements, five *cis*-elements (ARFAT, AUXREPSIAA4, ASF1MOTIFCAMV, GGTCCCATGMSAUR, and NTBBF1ARROLB), which are associated with auxin response and transcriptional activation, were identified in these *CiARF* promoters. To further investigate characteristic regions of *CiARF* promoter, the online MEME utility was employed to analyze the distribution of motifs in 19 *CiARF* promoters. A total of 15 distinct motifs were identified among 18 *CiARF* promoters and designated as motifs 1–15; the length of the motifs varied from 11 to 40 nucleotides (Figure S3). One of five motifs (motifs 1/2/3/5/7) was found in all 18 *CiARF* promoters, and motif 12 was found in only five *CiARF* promoters (Figure S3). However, these motifs have not yet been functionally characterized, and it remains to be investigated whether these motifs confer unique functional roles to *CiARF* genes.

### Expression of *CiARF* genes in different sweet orange tissues

**Figure 4:**
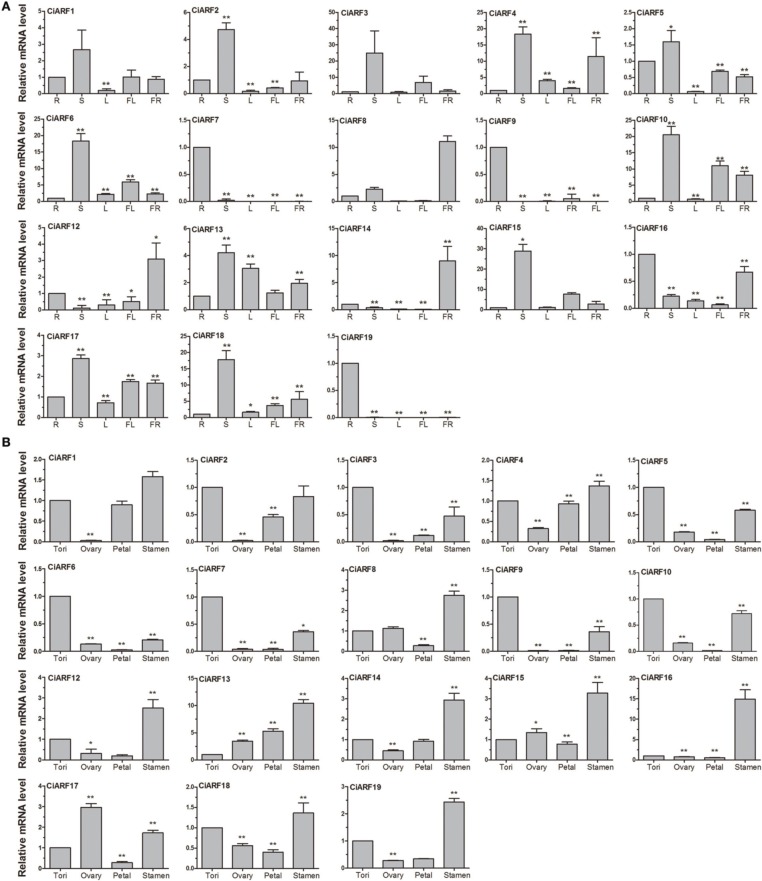
**The expression of *CiARF* genes in different sweet orange tissues**. **(A)** The expression of *CiARF* genes in root (R), stem (S), leaf (L), flower (Fl), and fruit (Fr) of sweet orange. **(B)** The expression of *CiARF* genes in floral organs of sweet orange, torus (T), ovary (O), petal (P), and stamen (S). Relative transcript levels are calculated by real-time PCR with β-*actin* as a standard. Data are means ± SE of three separate measurements.

To probe the physiological roles of *CiARF* genes, the temporal expression of individual members of the gene family was examined using real-time PCR. Transcript accumulation could be assessed for 19 *CiARF* genes in different tissues including root, stem, leaves, flower, and fruit (Figure 4A). The expression data showed a high variability in transcript abundance of the *CiARF* genes in various tissues and organs, strongly indicating the diversified functions of the *CiARF* genes in citrus growth and development. The *CiARF* genes, except *CiARF11*, could be detected in root, stem, leaf, flower, fruit, and floral organs using real-time PCR (Figure 4A). Some *CiARF* genes demonstrated organ/tissue-specific expression patterns in sweet orange. The expression of four *CiARF* genes (*CiARF7/9/16/19*) were highly expressed in sweet orange roots; *CiARF7/9/19* particularly exhibited root-specific expression in sweet orange (Figure 4A). The nine *CiARF* genes (*CiARF2/3/4/5/6/10/15/18*) were expressed more strongly in stem than in the other organs. *CiARF13* was relatively highly expressed in leaves compared with other tissues except stems. *CiARF8/12* were especially expressed in fruit. In general, most of the *CiARF* genes exhibited low expression in reproductive organs, but a high expression level in vegetative organs except leaves in this study (Figure 4A).

Expression of most CiARF genes could be observed in different tissues of the sweet orange floral organs (torus, ovary, petal, and stamen). In general, transcription of most *CiARF* genes was higher in the stamen than in other parts (Figure 4B). However, relatively higher expression levels of *CiARF17* were detected in the ovary compared with other tissues in this study, while six *CiARF* genes (*CiARF3/5/6/7/9/10*) were transcribed more strongly in tori than in other tissues (Figure 4B). In contrast, only *CiARF13* exhibited relatively high transcript levels in the petal except stamen (Figure 4B).

### Expression of *CiARF* genes during fruit ripening process of sweet orange

**Figure 5:**
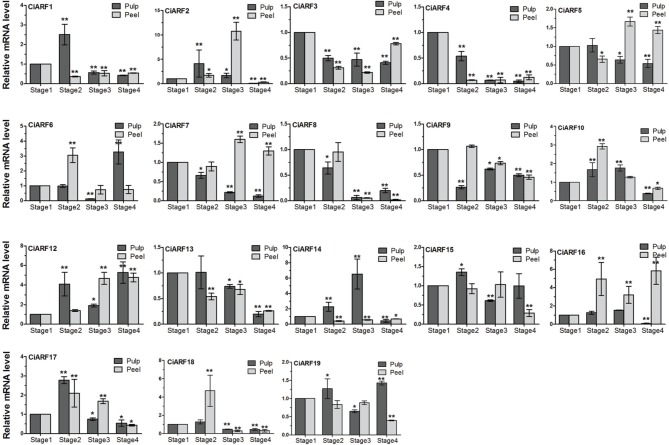
**The expression of *CiARF* genes during the fruit ripening process**. Real-time PCR was used to assess accumulation of *CiARF* gene at four developmental stages (stage 1: 170 DAF; stage 2: 200 DAF; stage 3: 230 DAF; and stage 4: 250 DAF) of sweet orange peel and pulp by real-time PCR, respectively. Relative transcript levels are calculated by real-time PCR with β-*actin* as a standard. Data are means ± SE of three separate measurements.

The high expression of *ARF* genes in fruit development, along with the previously reported role of auxin in controlling fruit ripening, prompted us to query the expression of *CiARF* genes during the sweet orange fruit ripening process (de Jong et al., 2009). To determine the expression dynamics, the transcript accumulation of *CiARF* genes was investigated at four developmental stages of sweet orange peel and pulp by real-time PCR, respectively. The results indicated that seven *CiARF* genes (*CiARF5/13/16/19* in peel and *CiARF9/15/19* in pulp) showed no significant difference from stage 1 to stage 3 (*p* < 0.001, Figure 5). These genes may play an important role in other development process. Six genes (*CiARF3/4/5/7/8/13*) and three genes (*CiARF4/8/13*) were down-regulated during the pulp and peel ripening process and most maintained low expression levels at stage 4 compared with stage 1, respectively. Two genes (*CiARF7/12*) were up-regulated during the peel ripening process (Figure 5). Six genes (*CiARF1/2/17/18* in pulp and *CiARF6, 18* in peel) were markedly up-regulated at stage 2 and then peaked at stage 3, followed by maintaining a low level of expression at stage 4 during the fruit ripening process. Interestingly, *s*ome *CiARF* genes (*CiARF1/2/17/18*, *CiARF4/7/13*, and *CiARF5/13*) exhibited a similar expression pattern of mRNA accumulation during the pulp ripening process (Figure 5). Similar expression patterns for several *CiARF* genes also indicate possible redundant functions during fruit developmental processes in sweet orange.

### Differential expression profiles of *CiARF* genes with hormone treatments

**Figure 6:**
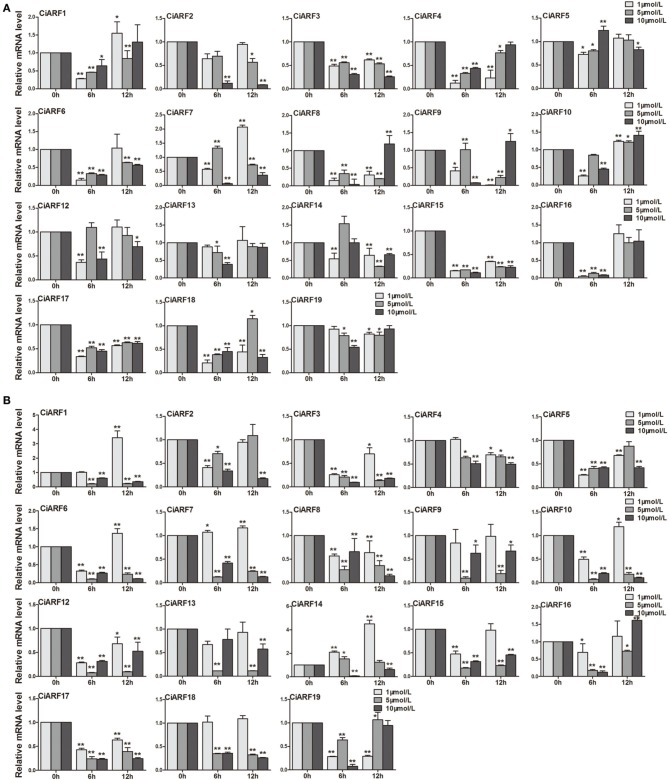
**The expression of *CiARF* genes in response to 1, 5, 10 μM IAA and NPA treatment, respectively**. Real-time PCR was used to assess accumulation of *CiARF* genes at 0, 6, and 12 h after treatment. Relative transcript levels are calculated by real-time PCR with β-*actin* as a standard. **(A)** The expression of *CiARF* genes by IAA treatment; **(B)** The expression of *CiARF* genes by NPA treatment. Data are means ± SE of three separate measurements.

Callus is an important material for citrus genetic transformation and tissue culture (Li et al., 2003). Meanwhile, auxin plays a very important role in the process of callus differentiation and plant regeneration (Pena et al., 2004). Thus, to determine the response of *CiARF* genes to exogenous auxin stimuli, their expression patterns in sweet orange callus at 0, 6, and 12 h were investigated after different concentrations of IAA and NPA treatments using real-time PCR (Figure 6). The results showed that nine *CiARF* genes (*CiARF1/3/4/6/8/15/16/17/18*) were suppressed at 6 h by three different concentrations of IAA treatment, and other three genes (*CiARF3/15/17*) were suppressed during whole treatment process (Figure 6A). The expression levels of four (*CiARF2/5/13/19*), six (*CiARF2/5/10/12/13/19*) and one (*CiARF1*) *CiARF* genes did not show significant changes (*P* < 0.01) by the 1, 5, and 10 μM IAA treatment, respectively (Figure 6A). On the other hand, the results indicated that nine *CiARF* genes (*CiARF1/3/6/7/10/12/15/17/18*) were suppressed during whole treatment process by the 5 μM and 10 μM NPA treatment (Figure 6B). The expression levels of five (*CiARF4/9/13/16/18*), two (*CiARF2/4*) and one (*CiARF9*) *CiARF* genes did not show significant changes (*P* < 0.01) by the 1 μM and 5 μM and 10 μM NPA treatment, respectively (Figure 6B). Interestingly, Interestingly, several CiARF genes followed a similar expression pattern after treatment by different hormones. For example, the expression patterns *CiARF3/15/16/17* by IAA treatment similar to that of *CiARF8/17* by NPA treatments (Figure 6).

From an applied perspective, we speculated that the experimental treatment may need a higher concentration. Therefore, the expression pattern of these *CiARF* genes in sweet orange callus at 0, 6, and 12 h were also investigated after 100 μM IAA and NPA treatments using real-time PCR (Figure S4). As expected, most of *CiARFs* were activated by IAA treatment. The expression levels of nine *CiARFs* (*CiARF2/5/8/10/14/15/16/18/19*) were increased at 6 h after the IAA treatment, and most of these genes were down-regulated at 12 h except *CiARF18*. It is worth mentioning that the expression of five *CiARF* genes (*CiARF1/3/6/9/12*) was markedly down-regulated just after IAA treatment (Figure S4). The response of *CiARF* genes to NPA treatment was also investigated in this study. The results indicated that six *CiARF* genes (*CiARF5/7/12/15/17/19*) were enhanced from 6 to 12 h under the NPA treatment, whereas six *CiARF* genes (*CiARF1/2/3/6/13/14*) were down-regulated at 6 h after NPA treatment (Figure S4). However, all down-regulated *CiARF* genes at 6 h after NPA treatment were up-regulated at 12 h.

## Discussion

The *ARF* gene family plays an important role during plant growth and developmental processes (Guilfoyle and Hagen, 2007). Therefore, to better elucidate the function of citrus *ARFs* in effecting specific auxin responses, the present study portrays the main structural features of the citrus *ARF* gene family. To isolate the complete array of *ARF* family members and perform expression profiling of these transcriptional regulators, this work took advantage of the recent sequencing of the citrus genome (Xu et al., 2013) and identified 19 *CiARF* genes (Table 1). There were fewer *CiARF* genes than *ARF* genes encoded in *Arabidopsis* (23), rice (25), and poplar (39) (Guilfoyle and Hagen, 2007; Kalluri et al., 2007; Shen et al., 2010). One of the reasons for the lower number of *ARF* genes encoded in the sweet orange genome could be that no large-scale duplication event occurred early in the evolution of the plant, unlike rice and *Arabidopsis* for which several such duplication events are known (Xu et al., 2013). Although the *CiARF* gene family overall has a lower number of genes than in *Arabidopsis*, two clades were larger in citrus. Clades III and IV contain six and four genes in sweet orange, respectively, but only five and three in *Arabidopsis* (Figure 1A). As an illustration of the wide diversification of ARF proteins in higher plants, the two clades were also larger in tomato (Zouine et al., 2014). The phylogenetic approach applied on a well-distributed set of plant *ARF* genes allowed identifying a specific subclass (subclass IV). Interestingly, this subclass contains a specific gene, *CiARF11*, which encodes a putative ARF protein that lacks the two protein–protein interaction domains (Figure 1C), known as domain III and IV, that are required for the binding to Aux/IAA proteins (Hagen and Guilfoyle, 2002). It is therefore likely that *CiARF11* escapes the the sequestration of ARF proteins through interaction with Aux/IAAs implicated in the classical mechanism of auxin signaling.

A large number of previous studies suggested that phylogenetic analysis enables functional prediction of various genes. For instance, phylogenetic analyses of the NAC families of citrus and *Arabidopsis* with their orthologs, whose functions are known in *Arabidopsis*, resulted in a nearly complete match between sequence conservation and functions or expression patterns (de Oliveira et al., 2011). Thus, a phylogenetic tree combining sweet orange and *Arabidopsis* ARF proteins would not only help elucidate the phylogenetic relationships of ARF proteins, but would also allow speculation on the putative functions of the sweet orange ARF proteins based on the functional clades currently described in *Arabidopsis* (Figure 1A). For example, CiARF3/4 grouped together with *Arabidopsis* AtARF3/4 into class I, referring to regulation of floral organs, developmental timing and patterning in *Arabidopsis* (Fahlgren et al., 2006; Finet et al., 2010). CiARF1/2/9/12/18 were assembled together with AtARF1/2/9/18 in class II, which represent the functional clades of the regulation of leaf senescence, floral organ abscission or auxin homeostasis (Ellis et al., 2005; Guilfoyle and Hagen, 2007). Similarly, AtARF5/6/7/8/19 were shown to play an important role in regulation of auxin-mediated morphogenesis, flower development or lateral root formation and gravitropism (Harper et al., 2000; Fukaki et al., 2006; Krogan et al., 2012). CiARF5/6/7/8/10/19 were grouped in class III with the five *Arabidopsis* proteins representing the functional clade with proteins responsible for similar functions during development in *Arabidopsis*. ARF10/16/17 are targeted by microRNA160 (miR160) in *Arabidopsis* (Liu et al., 2007). The other three proteins are regulatory factors related to pollen wall pattern formation, root cap formation, or seed germination (Mallory et al., 2005; Liu et al., 2007). In this study, four CiARF proteins (CiARF13/15/16/17) were grouped into these clades, which thus provided significant guidance to identify the citrus genes that play roles in the above process. Interestingly, class V had only contained one member from sweet orange (CiARF14). These results suggested the existence of citrus-specific ARF gene that were either lost in *Arabidopsis*, or acquired in the citrus lineages after divergence from the most recent common ancestor. These results further indicated that phylogenetic-based functional prediction might allow us to quickly select candidate genes, which could then be prioritized for further in functional analysis.

The features and number of domains present in the *ARF* sequences also provide useful information for the prediction of their functions in citrus (Guilfoyle and Hagen, 2007). In general, ARF proteins share three characteristic regions: the B3-type DNA binding domain in N terminal, the activation or repression domain in a middle region, and the homo- and heterodimerization domain in C terminal (Hagen and Guilfoyle, 2002; Guilfoyle and Hagen, 2007). In this study, protein sequence alignment of the CiARF proteins with their *Arabidopsis* counterparts confirmed that all had a typical ARF-type structure with a conserved B3-type DNA binding domain that consisted of a plant-specific B3-type subdomain, except CiARF11 (Figure 1C). In a comparison with the ARF members identified in other plants, those in sweet orange (21.05%) and *Brassica rapa* (22.58%) have a similar percentage of CTD truncated ARFs, while tomato has a higher rate of CTD-truncated ARFs (28.57%) (Guilfoyle and Hagen, 2007; Wu et al., 2011; Mun et al., 2012). There is evidence that the DBD is relatively less conserved and has experienced a rapid divergence during evolution (Romanel et al., 2009). In addition, all CiARF proteins contain a conserved putative monopartite nuclear localization signal at the end of the B3-type DNA binding domain similar to *Arabidopsis ARF* gene, except CiARF11 (Ulmasov et al., 1997). These nuclear localization signal was also predicted in *ARF* gene family of rice, and has recently been demonstrated to direct the gene product into the nucleus by a green fluorescent protein fusion assay (Shen et al., 2010). Thus, it is possible that these conserved motifs would be involved in the regulation of similar regulatory paradigms in different species.

Expression patterns of *CiARF* genes were investigated in different tissues using real-time PCR (Figure 4). Some *CiARF* genes showed organ/tissue-specific expression patterns in sweet orange. The expression patterns of *CiARF* genes suggest that the encoded proteins may perform both unique and redundant functions. One hypothesis regarding this phenomenon is that the distribution of specific motifs or specific patterns for a motif in proteins is associated with a specific clade in the phylogram; this model was supported by our data on CiARF proteins. These motifs may be involved in regulation of gene expression. In view of the presence of partial motifs and their distinct expression pattern, others believed that some *CiARF* genes, such as *CiARF11*, are pseudogenes. Its distinct gene structure also suggests that *CiARF11* is a pseudogene, and the data from real-time PCR confirms this suggestion. The information obtained on tissue-specific expression of the *CiARF* genes can be used to address the combinatorial usage of *CiARF* genes, allowing us to gain insight into the transcriptional program of different tissues, which is controlled by the *CiARF* genes. In previous studies, It has been reported that some ARF proteins modulate gene transcription during flower development in *Arabidopsis*, such as *AtARF3/6/8* (Nagpal et al., 2005; Pekker et al., 2005; Finet et al., 2010). However, it is worth noting most of *CiAFRs* (including *AtARF3/6/8*) show low expression in flowers in this study. There might be two possible explanations: at first, these genes may show high expression levels during the process of flower bud differentiation. The expression of these genes has been reduced when flowers at full bloom. The second possible explanation for this observation is that the regulatory mechanism of these genes differs between *Arabidopsis* and woody plants.

Auxin signaling is thought to play a key role in fruit ripening (de Jong et al., 2009). In this study, two *CiARF* genes (*CiARF1* in peel and *CiARF18* in pulp) appeared to be constitutively expressed in fruit ripening development (Figure 5), whereas the expression of other *CiARF* genes was transient, suggesting functional collaboration between these genes in fruit development. Since *ARF* genes are transcription factors that regulate auxin response genes, it would be interesting to determine the response of *CiARF* genes to IAA and NPA treatments. *Arabidopsis ARF4/5/16/19* and rice *OsARF1/23* transcripts have been reported to increase slightly in response to auxin, while *OsARF5/14//21* decreased marginally (Okushima et al., 2005; Wang et al., 2007). The transcript levels of most of the *CiARF* genes were up-regulated after 6 h by IAA and NPA treatment, and a high level of expression was maintained until 12 h for the NPA treatment. Compared with the NPA treatment, the CiARF proteins were down-regulated after 6 h and present at low levels at 12 h with the IAA treatment (Figure 6). Our promoter analysis identified five auxin signaling transduction-related *cis*-elements present in the promoter regions of most *CiARF* promoters. The diversity in the numbers and locations of their auxin signaling transduction-related *cis*-elements of *CiARF* genes may partially account for the different expression patterns of *CiARF*s under IAA and NPA treatment (Figure 6). Meanwhile, NPA is an auxin polar transport blocker (Lembi et al., 1971). The response and tolerance to NPA treatment of callus system might be remarkably different compared with plants. These results demonstrates the complexity of the IAA- and NPA-regulated expression of *CiARF* genes, and thus the relationship between auxin response elements and the change in expression of *CiARF*s by IAA and NPA treatments needs to be further investigated. In addition, although the roles of *CiARF* genes in these processes are not yet known, but given the genes' similarity to *Arabidopsis ARF* genes, the possibility of *CiARF* genes' participation in these different developmental processes in sweet orange cannot be excluded.

## Conclusion

The systematic characterization of the *ARF* gene family in citrus has revealed key features in the structures of the *CiARF* genes and in the relevant functions of this gene family in fruit growth and development of citrus. The expression patterns of the *CiARF* genes in various conditions will enable us to identify those that are expressed in a temporally regulated fashion. Studies of chromosomal distribution and phylogenetic of the *CiARF* genes have provided valuable insights on the evolutionary aspects of the citrus genome. The results of a comprehensive expression analysis of all identified *CiARF* genes under IAA and NPA treatment will help orient directions of molecular genetic studies, leading to better understanding of the functions of the *CiARF* genes in sweet orange and their future applications. The comprehensive identification and subsequent characterization of *CiARF* genes described here provide new insight regarding the potential role of some *ARF* genes in mediating citrus responses to auxin.

### Conflict of interest statement

The authors declare that the research was conducted in the absence of any commercial or financial relationships that could be construed as a potential conflict of interest.

## Supplementary material

Figure S1 **Characterization of 18 *CiARF* genes by gene ontology categories: (A) biological process; (B) molecular function; (C) cellular component**.

Figure S2 **Clade-wise distribution of conserved motifs in 18 CiARF proteins**. Fifteen putative conserved motifs were identified in the CiARF protein using MEME search tool (http://meme.nbcr.net/meme/). Different motifs are indicated by different colors. The length of motif in each protein represents the actual length, and motif sizes are indicated at the bottom of the figure.

Figure S3 **Clade-wise distribution of *cis*-motifs in 18 *CiARF* promoters**. Fifteen putative *cis*-motifs were identified in the upstream promoter region of *CiARF* genes using MEME search tool (http://meme.nbcr.net/meme/). Different motifs are indicated by different colors. The length of motif in each promoter represents the actual length, and motif sizes are indicated at the bottom of the figure.

Figure S4 **The expression of *CiARF* genes in response to 100 μM IAA and NPA treatment, respectively**. Real-time PCR was used to assess accumulation of *CiARF* genes at 0, 6, and 12 h after treatment. Relative transcript levels are calculated by real-time PCR with β-*actin* as a standard. Data are means ± SE of three separate measurements.
