# Supplementary figures and images for: Genome-wide identification, isolation and expression analysis of auxin response factor (ARF) gene family in sweet orange (Citrus sinensis)
Source: Front Plant Sci. 2015 Mar 30;6:119. doi: 10.3389/fpls.2015.00119 (PMC4378189; doi:10.3389/fpls.2015.00119)

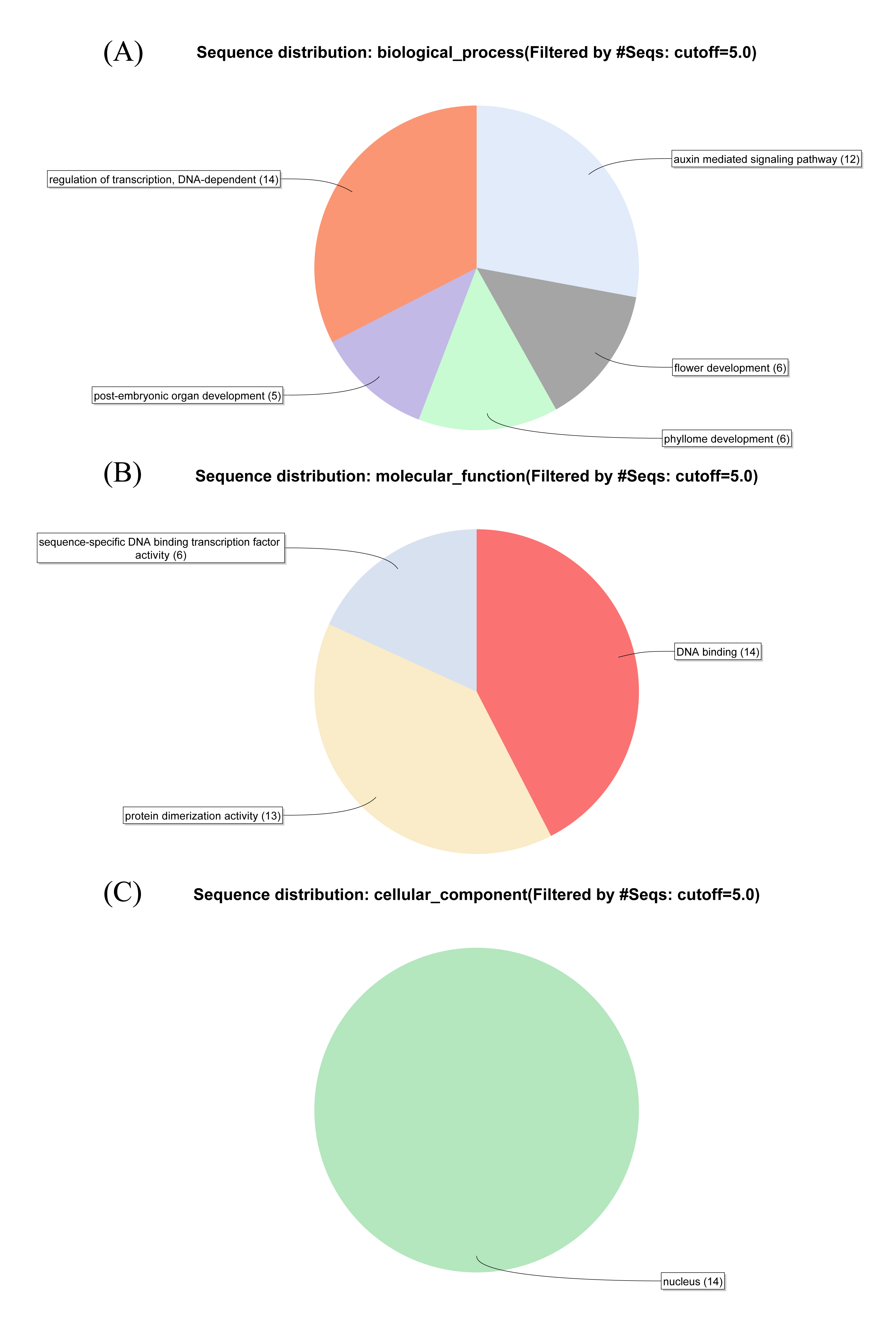

Supplement: Figure S1 — Characterization of 18 CiARF genes by gene ontology categories: (A) biological process; (B) molecular function; (C) cellular component. [file Image1.JPEG]

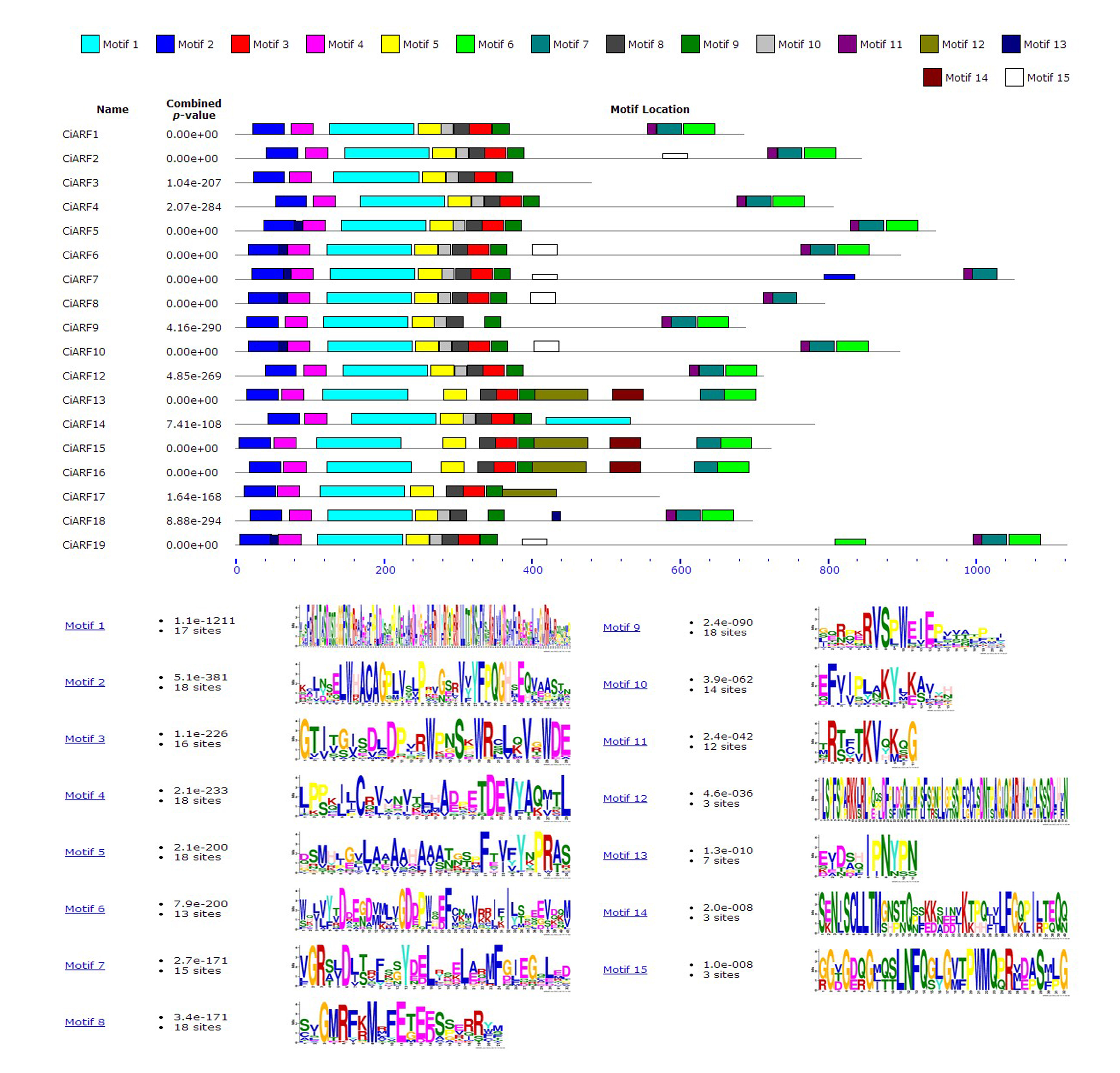

Supplement: Figure S2 — Clade-wise distribution of conserved motifs in 18 CiARF proteins. Fifteen putative conserved motifs were identified in the CiARF protein using MEME search tool (http://meme.nbcr.net/meme/). Different motifs are indicated by different colors. The length of motif in each protein represents the actual length, and motif sizes are indicated at the bottom of the figure. [file Image2.JPEG]

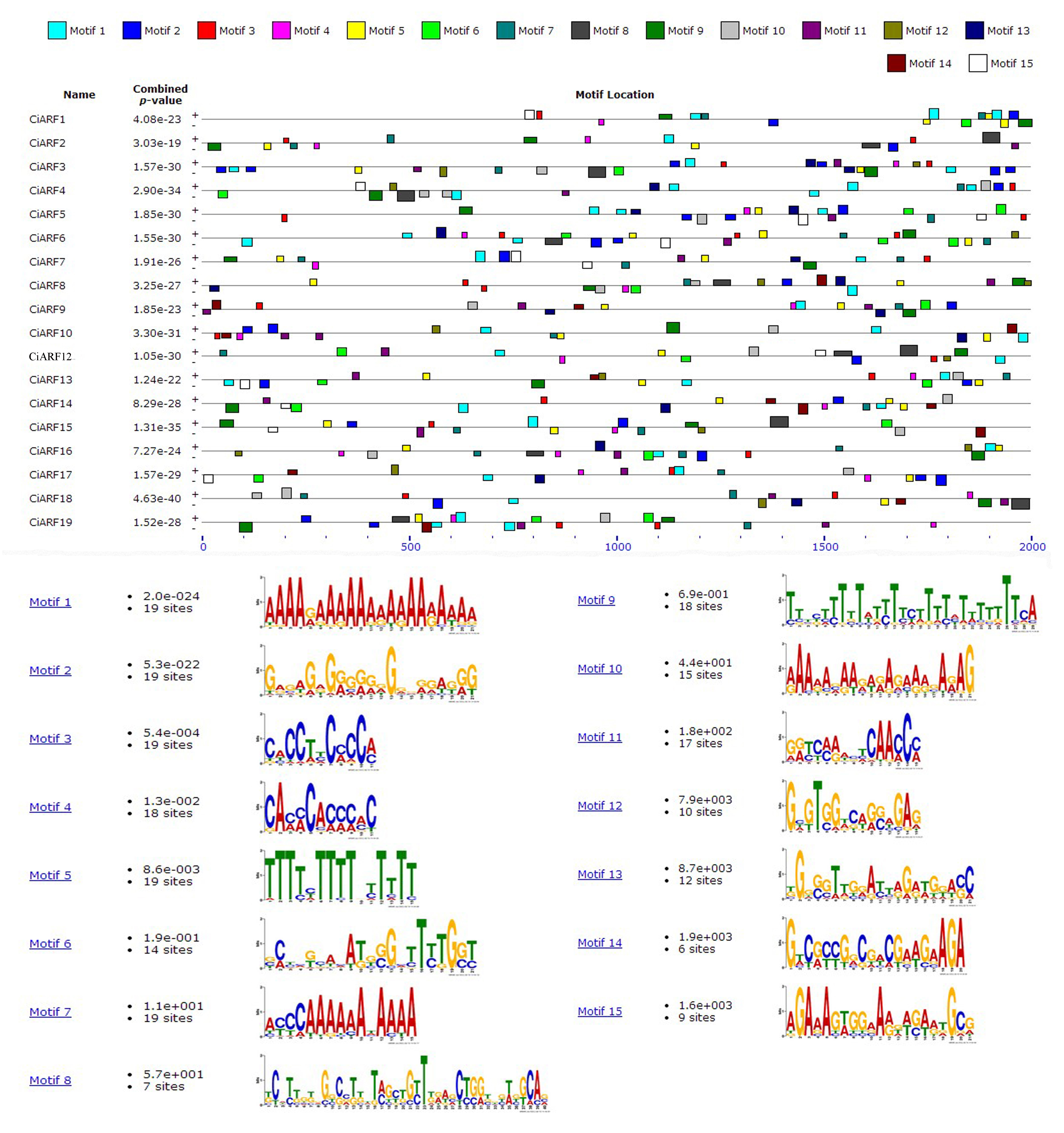

Supplement: Figure S3 — Clade-wise distribution of cis-motifs in 18 CiARF promoters. Fifteen putative cis-motifs were identified in the upstream promoter region of CiARF genes using MEME search tool (http://meme.nbcr.net/meme/). Different motifs are indicated by different colors. The length of motif in each promoter represents the actual length, and motif sizes are indicated at the bottom of the figure. [file Image3.JPEG]

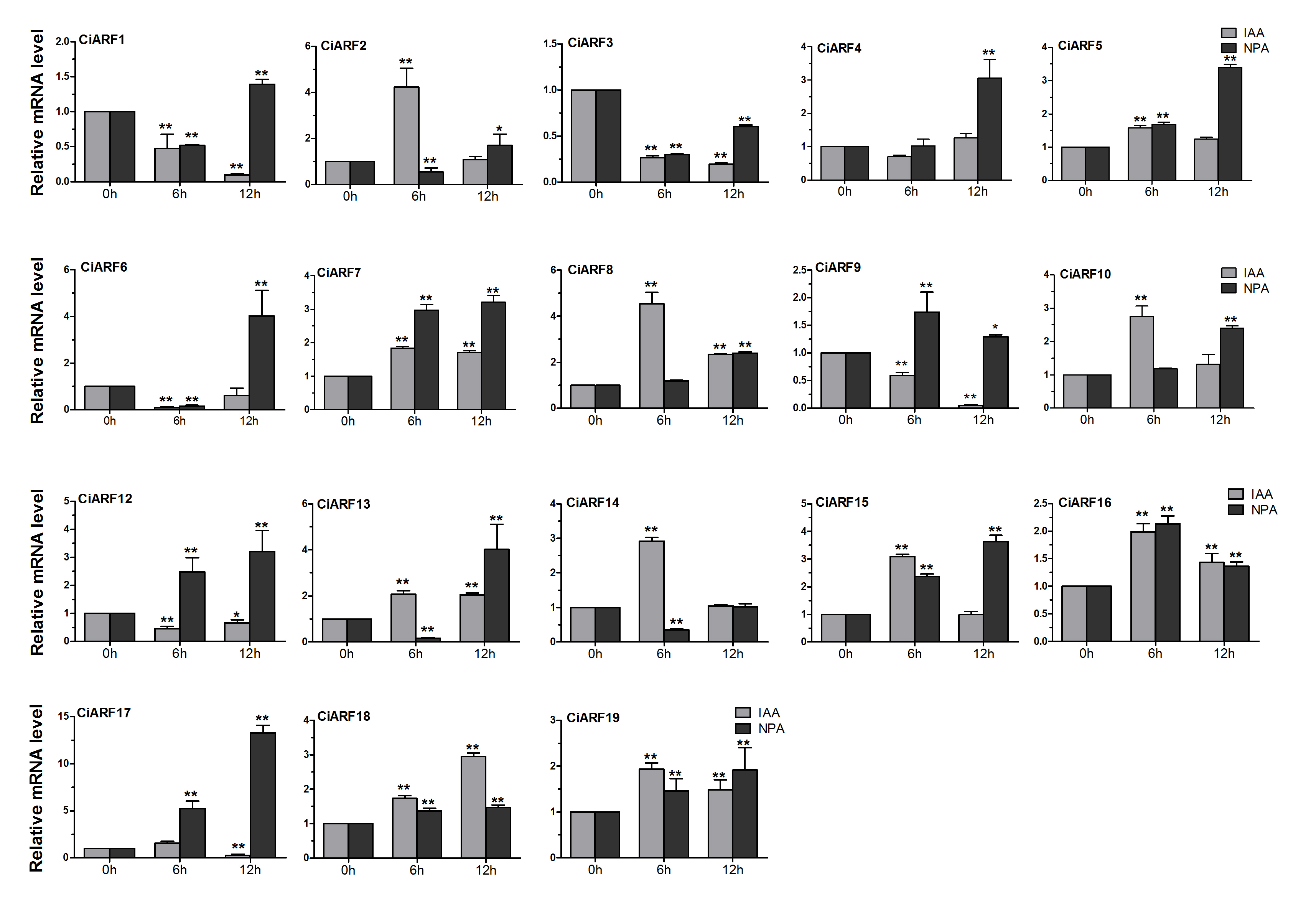

Supplement: Figure S4 — The expression of CiARF genes in response to 100 μM IAA and NPA treatment, respectively. Real-time PCR was used to assess accumulation of CiARF genes at 0, 6, and 12 h after treatment. Relative transcript levels are calculated by real-time PCR with β-actin as a standard. Data are means ± SE of three separate measurements. [file Image4.JPEG]
